# Mycophagy: A Global Review of Interactions between Invertebrates and Fungi

**DOI:** 10.3390/jof9020163

**Published:** 2023-01-26

**Authors:** Brianna Santamaria, Annemieke Verbeken, Danny Haelewaters

**Affiliations:** 1 Research Group Mycology, Department of Biology, Ghent University, K.L. Ledeganckstraat 35, 9000 Ghent, Belgium; 2 Faculty of Science, University of South Bohemia, Branišovská 31, 370 05 České Budějovice, Czech Republic; 3 Centro de Investigaciones Micológicas (CIMi), Universidad Autónoma de Chiriquí, David 0427, Panama

**Keywords:** biological control, fungal cultivation, fungivory, grazing, invertebrate ecology, literature data, microhabitats, secondary metabolites, spore dispersal

## Abstract

Fungi are diverse organisms that occupy important niches in natural settings and agricultural settings, acting as decomposers, mutualists, and parasites and pathogens. Interactions between fungi and other organisms, specifically invertebrates, are understudied. Their numbers are also severely underestimated. Invertebrates exist in many of the same spaces as fungi and are known to engage in fungal feeding or mycophagy. This review aims to provide a comprehensive, global view of mycophagy in invertebrates to bring attention to areas that need more research, by prospecting the existing literature. Separate searches on the Web of Science were performed using the terms “mycophagy” and “fungivore”. Invertebrate species and corresponding fungal species were extracted from the articles retrieved, whether the research was field- or laboratory-based, and the location of the observation if field-based. Articles were excluded if they did not list at least a genus identification for both the fungi and invertebrates. The search yielded 209 papers covering seven fungal phyla and 19 invertebrate orders. Ascomycota and Basidiomycota are the most represented fungal phyla whereas Coleoptera and Diptera make up most of the invertebrate observations. Most field-based observations originated from North America and Europe. Research on invertebrate mycophagy is lacking in some important fungal phyla, invertebrate orders, and geographic regions.

## 1. Introduction

Mycophagy (or fungivory) is the consumption of fungi by other organisms. This interaction has been documented in many groups such as bacteria [1], mammals [2], reptiles [3], birds [4], and invertebrates [5,6,7]. Mycophagy is common in almost all ecosystems where fungi and other organisms occur but does not receive as much attention as similar interactions. A Google Scholar search (on 23 December 2022) on “herbivory” yielded 316,000 results whereas “mycophagy” and “fungivory” only have 4840 and 1460 results, respectively.

**Figure 1:**
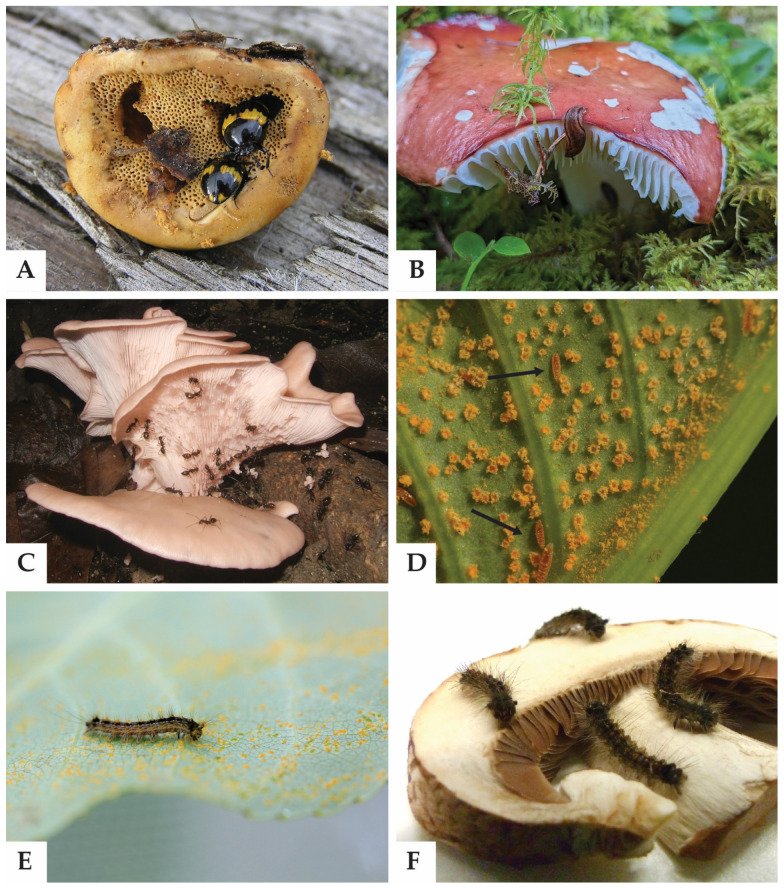
Examples of mycophagy in invertebrates. (**A**) *Diaperis boleti* tenebrionid beetles (Coleoptera) feeding on *Fomitopsis pinicola* (Polyporales, Agaricomycetes) in Białowieża Forest, Poland. Photo: Beentree, Wikimedia Commons. (**B**) A slug (Gastropoda) on *Russula* sp. (Russulales, Agaricales) in Stožec, Czech Republic. Photo: Michiel D. de Groot. (**C**) *Euprenolepis procera* ants (Hymenoptera) are known for their *Pleurotus*-harvesting (Agaricales, Agaricomycetes) behavior in southeastern Asia. Photo: Volker Witte, Wikimedia Commons. (**D**) *Mycodiplosis* midges (Diptera) (arrows) feeding on *Uromyces ari-triphylli* aecia (Pucciniales, Pucciniomycetes) in Minnesota, USA. Photo: iNaturalist observation #79142892 by davidenrique. (**E**) A gypsy moth caterpillar (*Lymantria dispar*, Lepidoptera) consuming spores of the rust fungus *Melampsora laricis-populina* (Pucciniales, Pucciniomycetes) on a leaf of black poplar (*Populus nigra*). Photo: Fransizka Eberl. (**F**) The same *L. dispar* caterpillars were also observed feeding from basidioma slices of *Agaricus bisporus* in laboratory setting (Agaricales, Agaricomycetes) (F. Eberl and S.B Unsicker, unpubl.). Photo: Fransizka Eberl.

Mycophagous interactions between fungi and mammals are relatively well studied, especially in the case where angiocarpic (i.e., truffle-like) and hypogeous (i.e., below-ground) sporocarps are collected and eaten by mammal dispersers. Invertebrates have thus far received less attention, although mushrooms frequently house larger or smaller invertebrates, such as snails, collembolans, insect larvae, et cetera (Figure 1). Invertebrates and fungi are indeed commonly found together in the same environments. Invertebrates in these environments are known to utilize fungi as a food source. Even though it has ramifications for agriculture and integrated pest management [8,9,10,11,12,13], invertebrate mycophagy is still an underexplored interaction.

To date, there has not been a global overview of mycophagous invertebrates and their fungal hosts. Many studies have focused on mycophagy in invertebrates, but their information is scattered. Early accounts of mycophagy have been casual observations of animals feeding on sporocarps (reviewed in [14]). One of the first comprehensive bibliographies of invertebrate mycophagy published in 1975 cited around 300 references concerning insects feeding on fungi [15]. In their book *Fungus–Insect Relationships: Perspectives in Ecology and Evolution*, Wheeler and Blackwell [16] presented four chapters containing lists of invertebrates and the fungi they feed on [17,18,19,20]. Several global reviews on mycophagy in Coleoptera were published [21,22,23], whereas papers on dipteran mycophagy have focused on a particular geographic region [24,25,26,27,28]. Recently, an extensive review of fungi associated with the mycophagous millipede *Brachycybe lecontii* in North America was published [6]. Insects inhabiting fungal sporocarps have been particularly studied in Sweden [29,30,31,32,33,34].

There are many examples of herbivorous invertebrate and plant interactions altering community structures through competition, defensive mechanisms, and mutualisms [35,36,37]. These same mechanisms are present in mycophagous invertebrate and fungal interactions. Mycophagy can influence community structure through positive interactions such as increased spore dispersal [38,39,40] or fungus-farming [41,42,43]. Similarly, negative interactions such as the destruction of fungi or death of the invertebrate through defensive compounds alter communities [44,45,46,47]. These interactions can also affect organisms outside the interaction by altering nutrient availability or influencing the outcome of competitive interactions [48,49,50]. These small-scale interactions can add up to larger, overall effects on entire communities. This can have important consequences for ecological studies that fail to take all total interactions into account.

The reason why mycophagy has received less attention than other forms of predation may be that fungi are in general understudied [51]. Current estimates of the true biodiversity of fungi range from 1.5 to 12 million whereas today only 148,000 species are described [52]. One consequence of the lack of research on fungi is that interactions between fungi and other organisms are also understudied. The aim of this review is to provide a comprehensive, global view of mycophagy in invertebrates to bring attention to interactions that need more research. 

## 2. Materials and Methods

Peer-reviewed papers were searched through the Web of Science Core Collection (https://www.webofscience.com/wos/woscc/basic-search, accessed on 28 September 2022) in two separate searches, using the terms “mycophagy” and “fungivore” in “All Fields”, respectively. Relevant articles that discussed invertebrates were included in a marked list. Studies were excluded if they did not mention an invertebrate or fungus classified to at least the genus level. Members of the fungus-like phylum Oomycota (the so-called pseudofungi *sensu* [53]) were included. Only articles in English or with English abstracts containing the needed information were included. To have more complete coverage of the research area, in-text citations in the listed articles were also included when they met the above criteria. The following information was extracted from the literature and added to a spreadsheet (Excel v. 2211): invertebrate species, fungal species, laboratory versus field observations, and geographic location of field observations (when applicable). Observations were marked as “field” when they took place without any outside influence and “laboratory” when they took place in an experimental setting. When invertebrates were collected and brought to the laboratory to examine their gut contents, the record was counted as a field observation. When a record included both laboratory and field observations, it was recorded twice. If the type of observation could not be determined the field was left blank [21,54,55]. In one paper, the geographic location field was left empty because we were unable to determine the exact location of the field observations [25]. Surveyed herbarium collections were classified as field observations [56,57]. Fungal species were updated to reflect the current taxonomy and their classification (family, order, class) following Index Fungorum [58]. Updated species were written as *New species* [as *Old species*]. Invertebrate classification follows the Global Biodiversity Information Facility [59]. Different variations, forms, and subspecies were ignored for both fungi and invertebrates. All figures and maps were created using the R software v. 3.6.3 [R Core Team, 2020] and the *ggplot2* package [60]. 

## 3. Results

**Figure 2:**
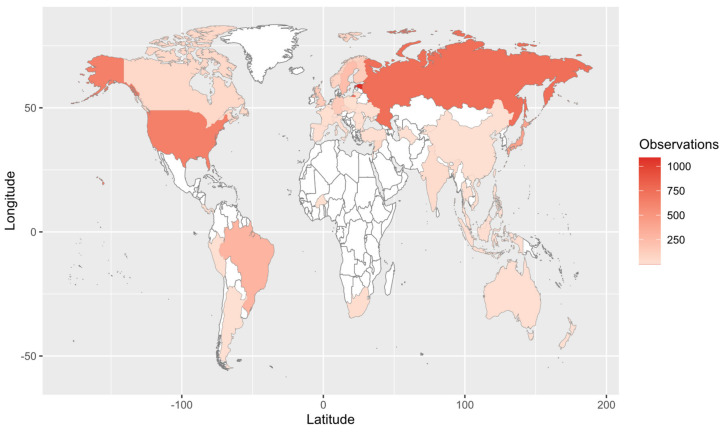
Map of field observations reported in the literature search. We reviewed 209 articles that comprised 6093 observations, of which 4287 are shown here (i.e., those with country-specific location information).

**Figure 3:**
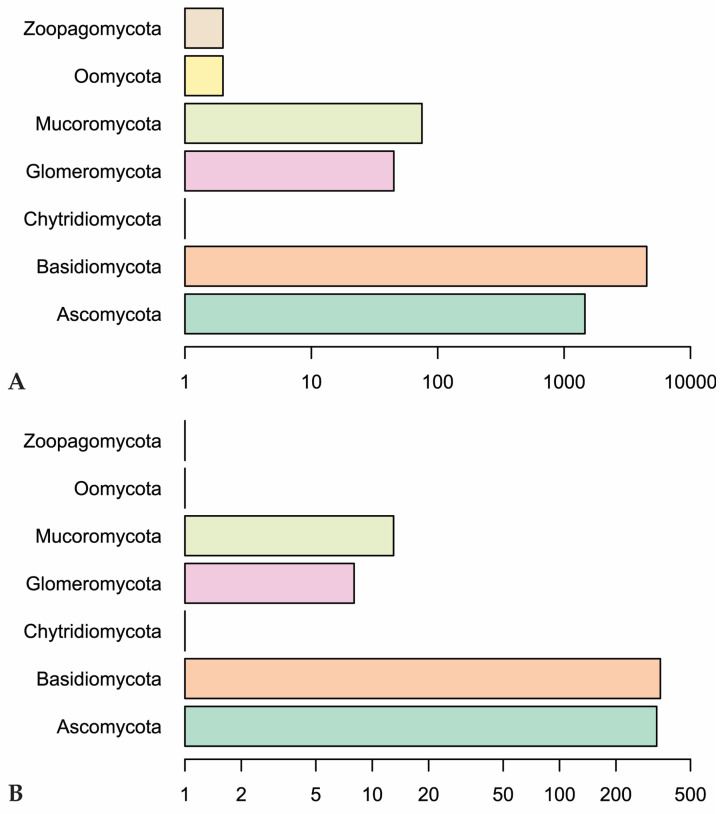
(**A**) Number of observations in fungal phyla in our dataset. (**B**) Genus-level diversity of represented fungal phyla. Scales are logarithmic.

**Figure 4:**
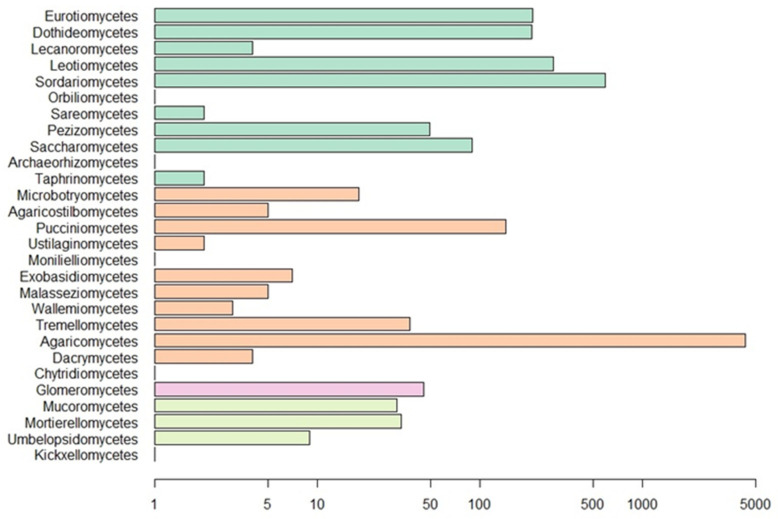
Number of observations for fungal classes in our dataset. Scale is logarithmic. Classes are colored by phylum as in Figure 3.

Our Web of Science search resulted in 153 papers. These primary papers combined with the in-text citations therein yielded 209 articles that we reviewed—including a total of 6093 observations (Table S1). Of those, 4287 are field observations with country-specific location information (Figure 2). Basidiomycota is the most represented phylum (4506 observations) in the literature, followed by Ascomycota (1462), Mucoromycota (75), and Glomeromycota (45) (Figure 3). In terms of generic diversity, Basidiomycota (345 genera) and Ascomycota (330) are very similar, followed at a great distance by Mucoromycota (13), Glomeromycota (8), Chytridiomycota, Oomycota, and Zoopagomycota (one each) (Figure 3). Of the fungal classes in our dataset, Agaricomycetes is represented the most (4282 observations), followed by Sordariomycetes (592) and Leotiomycetes (283) (Figure 4). Five genera (*Knufia*, *Pseudopenidiella*, *Sympodiella*, *Xenobotrytis*, and *Veronaea*) are currently not assigned to a class and placed into Ascomycota *incertae sedis*.

**Figure 5:**
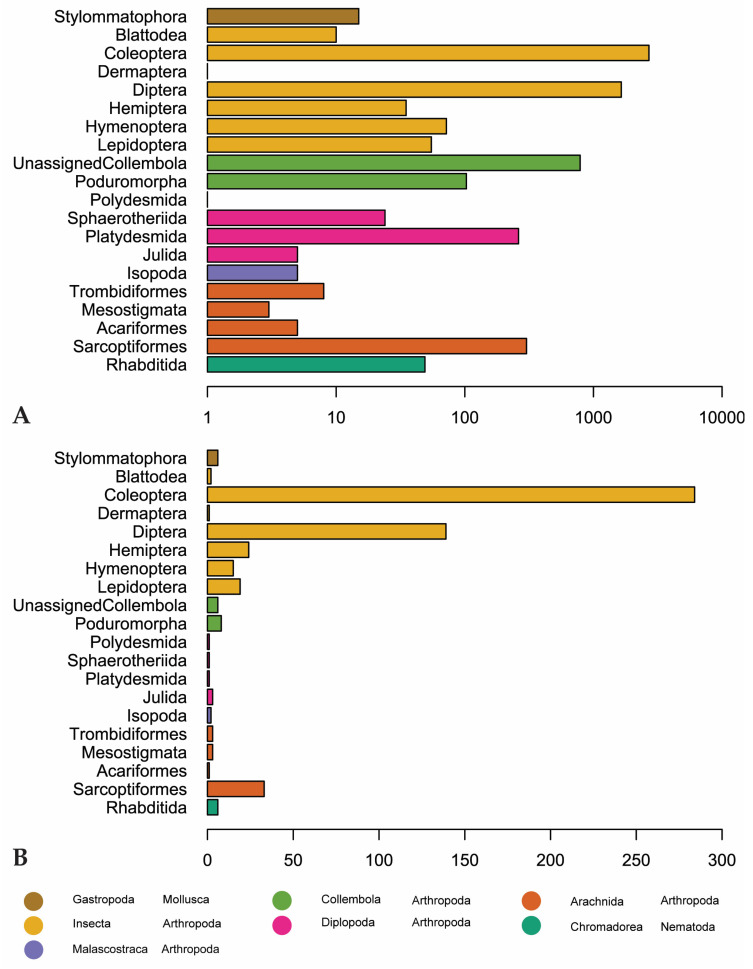
(**A**) Number of observations for invertebrate orders in our dataset. Scale is logarithmic. (**B**) Genus-level diversity of reported invertebrate orders. Orders are colored by class (Arachnida, Chromadorea, Diplopoda, Gastropoda, Insecta) or subclass (Collembola).

Coleoptera is the most recorded (2703 observations) and diverse invertebrate order (284 genera) followed by Diptera (1646 observations, 139 genera). Sarcoptiformes, within Acari, is the third-most recorded and diverse order, with 302 observations and 33 genera (Figure 5). “Unassigned Collembola” are observed more, but this is an assemblage of Collembola that have not been assigned to an order according to GBIF. There are 789 observations of this group, representing only 6 genera (*Entomobrya*, *Heteromurus*, *Orchesella*, *Pogonognathellus*, *Sinella*, and *Tomocerus*) (Figure 5).

## 4. Discussion

### 4.1. What Is the Diversity of Fungi Consumed by Invertebrates?

**Figure 6:**
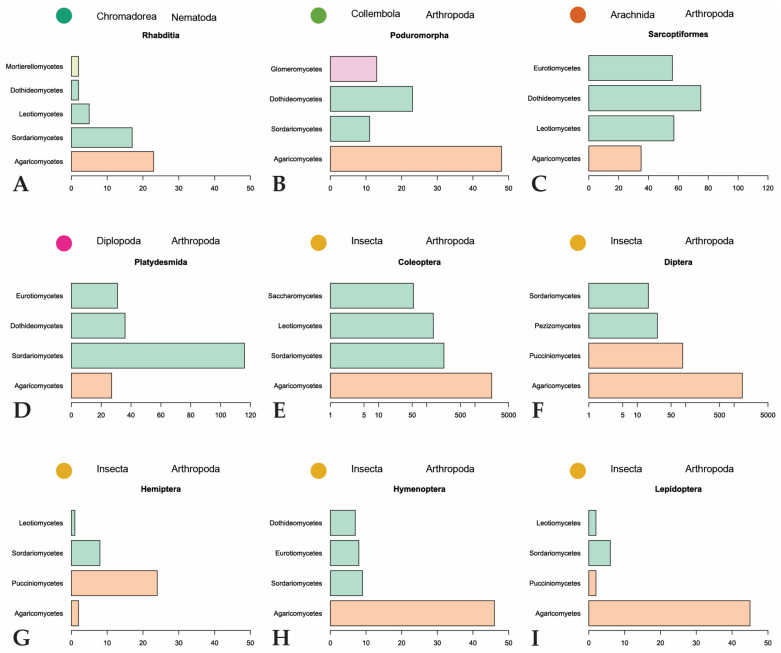
Summary of major invertebrate orders and representatives of the four most represented fungal classes they feed on. Fungal classes are colored by phylum as in Figure 3. (**A**,**B**,**G**–**I**): scales to 50; (**C**,**D**): scales to 120; (**E**,**F**): scales are logarithmic.

The majority of fungal observations are made in Agaricomycetes (Figure 6). This is a large, cosmopolitan class in Basidiomycota that contains many mushroom-forming fungi [61]. These macrofungi provide nutrition or a temporary home to many invertebrates. Every invertebrate group included in the dataset, with the exception of Dermaptera, is recorded feeding on Agaricomycetes fungi. A large diversity of dipterans are observed using these fungi as a habitat to breed, feed, and live in [26,32,62,63,64,65,66]. The same behavior is found among many different families of Coleoptera [21,29,62,67,68,69,70,71]. To a lesser degree, this behavior is also observed in Hymenoptera [29,32] and Lepidoptera [29,32,33,66]. Hemipterans, specifically representatives in the families Aphididae and Miridae, are hypothesized to inhabit sporocarps or sclerotia of Agaricomycetes [72,73,74]. The non-insect invertebrates utilize Agaricomycetes as food sources and not as microhabitats for breeding or development [75,76,77,78,79].

Despite it being the most diversified phylum of Fungi [80], Ascomycota is only the second-largest phylum represented in our dataset. Many of the recorded ascomycetous fungi belong to Sordariomycetes, followed by Leotiomycetes, Eurotiomycetes, and Dothideomycetes. Sordariomycetes is a diverse class of fungi that exist in many environments, including many plant and insect pathogens and endophytes [81]. The invertebrates largely associated with fungi in this class are Coleoptera, unassigned Collembola, and *Brachycybe lecontii* (Platydesmida, Diplopoda). The largest family of Coleoptera that feed on Sordariomycetes is Curculionidae. Some members of this family are known as ambrosia beetles and form complex mutualistic relationships with ambrosia fungi that are cultivated in fungal gardens in trees. Other members, bark beetles, cultivate pathogenic fungi in trees [82]. Ambrosia and bark beetles are widely studied because of their ability to spread fungal diseases [83,84,85]. The unassigned Collembola, which comprise the three families Entomobryidae, Orchesellidae, and Tomoceridae, feed on a wide diversity of fungi with different lifestyles in Sordariomycetes. This is consistent with the fact that Collembola are more generalist feeders that broadly feed on saprotrophic fungi but can also feed on mycorrhizal, parasitic, and lichenized fungi [79,86,87]. Similar to Collembola, *Brachycybe lecontii* is also a generalist feeder with a slight preference for fungi in the order Hypocreales [6].

Fungal phyla other than Basidiomycota and Ascomycota are represented far less in our dataset. Classes belonging to these phyla are scattered throughout the invertebrate orders, but none are reported more than the classes in the subkingdom Dikarya (Basidiomycota and Ascomycota). Mucoromycota are mainly plant-associated fungi that act as mycorrhizal partners, plant-associated parasites, or decomposers of organic material [88]. Glomeromycota is composed entirely of arbuscular mycorrhizal fungi [89]. Whereas fungi belonging to these phyla are expected to be present in many of the same environments as invertebrates, there are only 120 observations reported in our dataset. This could be because more research focuses on fungi belonging to Dikarya, especially taxonomic, phylogenetic, and interaction studies [90,91]. On top of this, it is difficult to identify non-Dikarya fungi in the environment to the level of genus, which would exclude them from our dataset. Laboratory studies are hindered by a lack of understanding of the conditions needed to culture many of these fungi. The non-Dikarya phyla are also much smaller in numbers than Basidiomycota and Ascomycota, so it is possible that they are less recorded in the dataset due to fewer representatives.

### 4.2. Why Do Invertebrates Feed on Fungi?

There are several benefits to mycophagy. The first is that fungi can represent an easily obtainable source of nutrients. Furthermore, sporocarps can serve as temporary shelters or breeding areas for invertebrates. In some cases, invertebrates evolve complex mutualisms in the form of fungal gardens to have a readily available supply of nutrients.

#### 4.2.1. Fungi as Food in the Environment

Fungi make up a large amount of biomass in many different environments and can be rich in digestible nitrogen depending on species [92,93]. These characteristics make them a viable food source. Invertebrates can be either mono- or oligophagous, meaning that they selectively feed on only one or a few fungal species, or polyphagous, feeding on multiple species of fungi. In general, invertebrates that feed on long-lasting Polyporales (bracket fungi) tend to be mono- or oligophagous whereas those that feed on ephemeral (i.e., short-lived) Agaricales are more polyphagous [33,94]. For non-sporocarp-forming fungi, the preference is highly dependent on invertebrate species, fungal species, as well as the environment in which they occur [6,95,96,97].

Two hypotheses have been posited to explain polyphagy in mycophagous invertebrates: the quantity and quality hypotheses [98]. The quantity hypothesis states that polyphagy is due to the low predictability of sporocarps. Sporocarps are often ephemeral and restricted to certain times of the year, making it difficult for invertebrates to reliably utilize one or two fungal species, meaning it is more beneficial to feed on multiple species. The quality hypothesis says that differences in chemical traits among fungi are not expected to be a major barrier to invertebrates. Polyphagous invertebrates should not be hindered by chemical differences among fungal species if they are to feed on multiple fungi. The quality hypothesis is also relevant to monophagy; host specificity to Polypores may be due to chemical barriers among species, which would encourage specialization on one or two species [33]. 

Multiple authors have tested the validity of these hypotheses. One study explored the insects feeding on sporocarps in different stages of development and decay. Fungal genera only had a small effect on the structure of the insect community (10-19%), providing support to the quality hypothesis as these genera represented different chemical traits [65]. However, invertebrates thought to be polyphagous may instead be oligophagous. As an example, Drosophilidae preferentially feed on a few fungal genera while occasionally feeding on other fungi [63,99]. Feeding on less preferred fungi could be a trade-off between the quality and quantity hypotheses [99].

In addition to purely serving as food sources, fungi may provide other benefits to invertebrates. As an example, *Littoraria irrorata* sea snails (Gastropoda) feed on *Spartina alterniflora* leaves but fungi also contribute to their diet, mostly *Typhicola typharum* (Pleosporales, Dothideomycetes) [100]. It is thought that fungi play three different roles in the diet of this species and other detritivores: (i) as a food source, (ii) by producing enzymes to help digest plant compounds, and (iii) by providing lipids essential for development [101].

#### 4.2.2. Fungi as a Microhabitat

Many invertebrates use fungal sporocarps as an environment to breed and lay eggs. This is most extensively studied in Coleoptera and Diptera although there are many other invertebrate groups that can live in sporocarps [30,62,66]. Diptera can preferentially utilize sporocarps that are fresh or dead although a majority choose decaying fungal material [25,65]. Depending on the age of the sporocarp, some Diptera can delay their development until decay starts [25]. Most Diptera show a lower degree of host specificity for sporocarps when compared to Coleoptera [25,33,65,102,103]. This trend may be explained by the fact that Diptera often prefer ephemeral sporocarps of Agaricales whereas Coleoptera that inhabit sporocarps mainly utilize long-lasting Polyporales.

Older studies traditionally reared organisms inside the sporocarps and used morphology-based identification to determine what was present. More recent studies use DNA metabarcoding to look at the structure of invertebrate communities in sporocarps. The first study based on DNA metabarcoding to study host-specificity in sporocarp-inhabiting invertebrates found a low level of specificity and little impact of fungal taxonomy on invertebrate communities [104]. Recently, Lunde and colleagues [105], using the same methods to test the influence of living fungal sporocarp traits on the assemblage of arthropods that reside in them, found that softer sporocarps housed more flies. Tougher sporocarps contain larger amounts of Acari and Coleoptera. Conversely, almost all arthropods were specific to one or two fungal hosts. The difference between the findings of these two studies is most likely due to the fungal species selected. The first study focused on fleshy agarics whereas the latter one included a broader range of fungi with different traits.

Sporocarp host specificity in invertebrates is a topic that is in need of more exploration. As technology advances, researchers can utilize new cost-effective and efficient ways to identify invertebrates in sporocarps. The shift away from traditional rearing techniques to DNA metabarcoding will enhance our understanding and give us new insights into this subfield [104,105,106,107,108,109].

#### 4.2.3. Fungal Cultivation

Some mycophagous invertebrates evolved complex mutualisms with fungi in the form of fungal cultivation. Fungal agriculture evolved in three groups of insects: ants, beetles, and termites. These mutualisms represent one of the most studied fungi-invertebrate relationships. Fungus-growing is hypothesized to have started in wood-boring weevils, then evolved in termites and ants [5]. Fungus-growing weevils (known as ambrosia beetles) are largely associated with fungi in the orders Microascales and Ophiostomatales (Sordariomycetes) [55,110]. Fungus-growing termites exclusively utilize fungi in the genus *Termitomyces* (Agaricales, Agaricomycetes) [111]. Finally, fungus-growing ants utilize mycelia and masses of yeast mostly in the tribe Leucocoprini (Agaricales, Agaricomycetes) [112]. More detailed studies and reviews on these mutualistic relationships can be found elsewhere in the literature [5,110,112,113,114,115,116].

### 4.3. How Do Mycophagous Interactions Affect Fungal Communities?

The species composition of fungal communities can be influenced by their interactions with invertebrates that feed on them. These interactions can be positive, for example when fungal propagules are dispersed by invertebrates, or negative, when overall fungal fitness is reduced [117]. Fungal mycophagy by invertebrates can also have consequences for organisms outside of the primary interaction, such as plants or other fungi [118,119,120,121]. 

#### 4.3.1. Spore Dispersal

Spore dispersal is one of the most significant ways in which animals influence fungal communities. Both invertebrates and vertebrates can disperse viable fungal spores through mycophagy [2,38,122,123]. This has been observed in many invertebrate groups.

Mycophagy has been hypothesized to be the primary means of spore dispersal in hypogeous fungi [124]. Hypogeous fungi are those that produce sporocarps partially or completely below-ground. More commonly known as truffles and false truffles, they reside in several phyla and classes of fungi. Fogel and Peck [124] recorded 36 coleopterans feeding on hypogeous fungi, some of them having viable spores in their fecal matter. Molluscs have also been shown to carry viable spores of hypogeous fungi [125].

Invertebrates can carry spores on their legs or in their intestines [38,126,127]. *Guyanagaster necrorhizus* (Agaricales, Agaricomycetes) spores adhere to the exoskeleton of termites in the genera *Cylindrotermes*, *Dihoplotermes*, and *Nasutitermes* [128] Several studies show that especially Diptera are adept at carrying viable fungal spores in their intestines [39,129,130,131]. These spores retain the ability to form mutualistic associations with plants [40,126] or infect organisms [132]. The viability of spores has been suggested to depend on several characteristics, implying variation in their ability to resist digestive enzymes [39]. However, the associations between the viability of spores on the one hand and pigmentation, thickness, and ornamentation of spores on the other are likely complex and not understood well.

Spores that are large, melanized, and have a thick, heavily ornamented outer wall are hypothesized to be adaptations “designed” for mycophagy and dispersal [133]. However, this idea has not been formally tested or proven. For example, contrary to this hypothesis, species of *Rhizopogon* and *Suillus* (Boletales, Agaricomycetes) have generally thin-walled, smooth, and hyaline spores yet with high rates of viability [131,134,135,136,137]. One final note to consider here is that invertebrates may be more efficient in spore dispersal compared to many mammals [125]

Fungi can be dispersed by multiple invertebrates. *Epichloë typhina* (Hypocreales, Sordariomycetes) is a plant pathogen known to cause choke disease in orchardgrass [138]. *Phorbia phrenione* (Diptera) fertilizes (i.e., it forms a dikaryotic phase through spermatization) *E. typhina* using a highly specific behavior [139]. *Phorbia phrenione* is known to only feed on *E. typhina* and is thought to be the primary vector for its spermatia [139]. However, several other species are also known to feed on *E. typhina* and potentially spread viable spores. *Arion subfuscus*, *Deroceras reticulatum*, and *Prophysaon andersoni* (Gastropoda) feed on *E. typhina* in grass fields. All three slug species excrete viable spores that can fertilize fungi and initiate infections [140]. In addition, *Aniulus bollman* (Diplopoda) may exploit *E. typhina* by feeding on it before it sporulates [141]. It is unknown whether *A. bollman* negatively affects populations of *E. typhina*. 

Ambrosia and bark beetles have specialized structures known as mycangia that can transport viable fungal spores to host trees and across generations [142]. Inside host trees, these beetles construct fungal galleries and cultivate fungal symbionts. The size, shape, and microbial communities of these mycangia are unique to each species [143]. The fungi these Coleoptera carry can cause destructive diseases in trees [144,145]. Similarly, some species of Siricidae wood wasps (Hymenoptera) also carry fungal spores in mycangia that are used to rot wood in trees and create a habitat for larval development [146,147].

Overall, spore dispersal is an important interaction between invertebrates and fungi. Through mycophagy, spores stick onto invertebrates or enter their digestive tract. Spores often remain viable and can be spread to germinate on their preferred host or used to fertilize other fungi. Whereas the phenomenon of pollen dispersal has been extensively studied in plants, fungal spore dispersal is still underexplored. Vašutová and colleagues [123] found only 33 articles that experimentally proved the successful transport of mycorrhizal fungi by animals. Of those, only nine studies have focused on invertebrates (without mentioning species numbers) despite evidence that many of them can carry viable spores [123]. In contrast, at least 40 mammal species have been experimentally shown to disperse viable spores through their scats [2]. 

#### 4.3.2. Grazing by Invertebrates

While mycophagy can provide benefits to fungi such as spore dispersal, it can also have negative effects. Mycophagy can decrease fungal fitness, as hypothesized for *Aniulus bollman* feeding on *Epichloë typhina* before it sporulates [141]. A study found that two Ciidae beetles, *Cis boleti* and *Octotemnus glabriculus*, decrease the reproductive fitness of their preferred fungal host, *Trametes versicolor* (Polyporales, Agaricomycetes) [68]. *Zearagytodes maculifer* (Coleoptera) reduces the germination of *Ganoderma applanatum* (Polyporales, Agaricomycetes) spores through feeding [148]. The overall effect of these interactions can alter competition among fungi occupying the same area by preventing one species from dominating others in what is referred to as top–down control.

Microcosm experiments have been frequently used to test how mycophagy affects soil fungal competition. In the absence of grazers, *Resinicium bicolor* (Hymenochaetales, Agaricomycetes) outcompetes both *Hypholoma fasciculare* (Agaricales, Agaricomycetes) and *Phanerochaete velutina* (Polyporales, Agaricomycetes) [149]. When allowed to graze on these fungi, *Oniscus asellus* (Isopoda) selectively feeds on *R. bicolor*, preventing it from outcompeting *H. fasciculare* and *P. velutina*. In a different scenario, *Panagrellus redivivus* (Nematoda) stimulates the growth of *H. fasciculare* through grazing, allowing it to escape exclusion by *R. bicolor* through a process called “gross mycelial contact” [150]. A similar experiment was conducted using the same three fungi at different temperatures. At higher temperatures and in the absence of grazers, *R. bicolor* dominates *H. fasciculare* and *P. velutina* [151]. When grazers are present, they preferentially feed on *R. bicolor* and decrease its ability to exclude *H. fasciculare* and *P. velutina* at higher temperatures. 

Crowther and colleagues tested the strength of top–down grazing on soil fungal community structures [152]. Grazing by *O. asellus* reduces the presence of dominant Basidiomycota fungi and increases both the presence of Ascomycota and Zygomycota through top–down control. In the rhizosphere, invertebrate grazing does not induce top–down control but instead stimulates growth in less dominant fungal taxa, contrasting to most other studies [153]. A meta-analysis on grazing in soil fungi found that the effects of grazing are highly dependent on the species of both fungi and invertebrates [50]. 

Fungal communities are made up of multiple species of fungi that interact with invertebrates and with each other in complex ways. It is difficult to apply the outcomes of these microcosm interactions to a natural environment. It is likely that multiple factors combine to influence the effect of grazing on fungal communities. Current studies are working to disentangle these factors [105,154,155,156], but it is still a field that requires more research.

### 4.4. What Is the Role of Secondary Metabolites in Mycophagy?

In plants, secondary metabolites (SMs) can be used to attract symbionts or deter predators. Fungi have similar SMs that can deter or attract mycophagous invertebrates. Although there are some widely recognized fungal SMs (e.g., penicillins and statins), there are still many that are relatively unknown, particularly ones that are involved in interactions with invertebrates [157,158,159].

#### 4.4.1. Deterrents

Secondary metabolites are hypothesized to have evolved as a defense against mycophagous animals [44,160]. The first study to experimentally demonstrate that fungi can use SMs as an antifeedant utilized *Aspergillus nidulans* (Eurotiales, Eurotiomycetes) and *Folsomia candida* (Collembola) [44]. *Folsomia candida* is a blind, soil arthropod that relies on chemical cues to find food. When offered wild-type *A. nidulans* and modified *A. nidulans* lacking SMs, *F. candida* preferentially consumes modified *A. nidulans* and shows a higher reproductive fitness compared to when it only preys on wild-type *A. nidulans* [44]. Similarly, *F. candida* prefers fungi without repellant crystalline structures or SMs on the hyphae and has a higher reproductive fitness when feeding on fungi without chemical defenses [45].

Fungi show induced resistance against grazing, i.e., showing a heightened defensive state after stimulation. For example, when fungal resistance genes are triggered in *A. nidulans* due to grazing, the fungus causes 100% mortality in *Drosophila melanogaster* (Diptera) larvae after feeding [46]. Collembola feed less on fungi that have been previously grazed on before [161]. The intensity of grazing can affect the induction of SMs. Low-intensity grazing by *O. asellus* isopods on *A. nidulans* does not have any effect on the expression of SM genes when compared to ungrazed *A. nidulans*, whereas high-intensity grazing leads to a lower expression of SM genes [162]. These results suggest that fungi may benefit more from putting energy into repair instead of costly defense compounds. High-intensity grazing could be also used as a manipulation tactic by invertebrates to reduce fungal defenses [162]. This tactic has been demonstrated experimentally; *Folsomia candida* foraging in high densities on *A. nidulans* shows a higher fitness when compared to low-density foraging [163]. 

It is possible for invertebrates to develop resistance to fungal SMs. However, both the mortality and overall fitness of *D. melanogaster* bred to be resistant to *A. nidulans* are reduced when exposed to the fungus when compared to a non-resistant strain [164]. This result shows a clear trade-off between fungal resistance and other survival traits. As such, it may not be as beneficial for mycophagous invertebrates to develop complete resistance to fungal SMs, but instead adopt behaviors that increase survival such as high-density feeding (*sensu* [162,163]).

There are still many important areas regarding antagonistic fungal SMs. Kempken and Rohlfs [165] highlighted key questions that remain unanswered. Experimental data have been gathered to shed light on some of these questions, but there are still important ones that remain unanswered such as the ecological impact of fungal SMs [166]. In the past few years, some studies have been published about SMs derived from entomopathogenic fungi particularly [167]. There might be some overlap between these and SMs from fungi involved in invertebrate mycophagy. 

#### 4.4.2. Attractants 

Analogue to plants sending signals to attract pollinators, fungi have evolved SMs to attract mycophagous invertebrates for spore dispersal and other mutualisms. Fungal SMs that attract invertebrates are thought to have evolved from those that were originally used for defense [168]. In addition, insect attractant signals in yeasts are an ancient, evolved trait that arose before angiosperms [169]. 

There are many examples of fungi attracting mycophagous invertebrates. *Tuber* species (Pezizales, Pezizomycetes) produce dimethyl sulfide that attracts specialist arthropods such as Diptera, Staphilinidae (Coleoptera), and Tineidae (Lepidoptera) [170]. *Tyrophagus putreseentiae* (Acari) spreads viable fungal spores and is strongly attracted to SMs produced by 15 different species in Agaricomycetes [171,172]. Species of *Scheloribates* (Acari) are strongly attracted to SMs released by different species of fungi [173]. *Scheloribates* species can carry fungal spores in their digestive tracts, but the viability of these spores has not been evaluated [174]. Volatiles are released by yeasts to attract *D. melanogaster*, and these compounds are mimicked by the monocot *Arum palaestinum* [175].

Fungal preference in mycophagous invertebrates is influenced by SMs. This leads to the idea that SMs might be used by less attractive fungi to attract invertebrates. In a food preference experiment, *Onychiurus armatus* (Collembola) were presented odors from *Metapochonia bulbillosa* (Hypocreales, Sordariomycetes), *Penicillium spinulosum* (Eurotiales, Eurotiomycetes), and *Umbelopsis isabellina* (Umbelopsidales, Umbelopsidomycetes) [176]. Odors emitted by *P. spinulosum* and *U. isabellina* were indistinguishable from *O. armatus*, but they preferred to feed on *U. isabellina* when allowed to taste the fungi [177]. Preferences also changed depending on whether the fungi were grown on agar or soil.

Fungi can form dramatic structures to attract invertebrates. Pseudoflowers are produced by parasitic fungi that infect a plant and force it to create structures resembling flowers. These structures release SMs attracting pollinators. Compared to the plant’s own flowers, the pseudoflowers have higher sugar contents to keep the pollinators for a longer period of time [178]. The pseudoflowers formed by *Fusarium xyrophilum* (Hypocreales, Sordariomycetes) are composed entirely of fungal material and release SMs to attract pollinators [179]. Rust fungi can also produce pseudoflowers that attract and trick invertebrates into pollinating them [180,181]. Whether pseudoflowers are classified as mycophagy is unclear in the literature.

Secondary metabolites can be used to attract and then trap or kill invertebrates. When ingested, *Nidulariopsis iowensis* and *Sphaerobolus stellatus* (Geastrales, Agaricomycetes) cause lethargy and the eventual encapsulation and immobilization of nematodes [182]. *Climacodon septentrionalis* (Polyporales, Agaricomycetes) also immobilizes mycophagous nematodes but unlike *N. iowensis* and *S. stellatus*, it consumes the nematodes after immobilization [183]. Additionally, *Pleurotus* species have antagonistic effects on nematodes [184]. *In-vitro* experiments revealed that nematodes touching droplets of fungal toxin show a dramatic response resulting in immobilization after as little as 30 s [185,186]. Some of these fungi use odors that mimic nematode cues to attract them [187]. This behavior is thought to have evolved as a way for fungi to make use of an alternative source of nitrogen in nitrogen-poor environments, but there still remain many questions about the evolution between these fungi and the nematodes they prey on [188,189].

Attractants in yeasts have been the most extensively studied compared to other fungal groups [169,190,191,192]. Even so, important questions such as how different compounds influence invertebrate behavior remain unanswered [193]. A recent review has shown that many SMs are known, but their function in the ecosystem needs clarification [194].

### 4.5. How Can Mycophagy Be Applied to Agriculture?

Both mycophagous invertebrates and fungi play prominent roles in agriculture. Mycophagous invertebrates can spread fungal diseases to agriculturally important plants [8,9,10,195]. Invertebrates can act as pests on commercially grown fungi [196]. They may also be developed as biological controls for fungal diseases [11,12]. Alternatively, there is potential to use fungi engaging in mycophagy to control invertebrates [13,197]. 

In some cases, mycophagy can interfere with fungi used as biocontrol for other fungi. *Aphelenchoides* nematodes feed on and reduce the efficacy of *Trichoderma harzianum* (Hypocreales, Sordariomycetes) as a biocontrol agent against the soil pathogen *Sclerotinia sclerotiorum* (Helotiales, Leotiomycetes) [198]. *Trichoderma harzianum* can persist in the sclerotia of *S. sclerotiorum*, protecting it from predation [199]. However, *Aphelenchoides* nematodes have been shown to penetrate inside *S. sclerotiorum* sclerotia and potentially kill *T. harzianum* [200]. *Sclerotinia sclerotiorum* itself has been considered for use as a biocontrol agent against invasive weeds [201,202]. In controlled experiments, its efficacy as a biocontrol against the invasive asterid *Centaurea stoebe* is dependent on when *A. saprophilus* and *T. harzianum* were introduced [203]. These interactions highlight the potential consequences that mycophagous invertebrates have in integrated pest management. 

Grazing by mycophagous invertebrates on mycorrhizal fungi can help encourage plant growth. Low-density grazing by *O. armatus* enhances the biomass and colonization rate of the fungus *Paxillus involutus* (Boletales, Agaricomycetes) as well as the nitrogen uptake by its ectomycorrhizal partner, *Pinus contorta* [204]. Collembola especially feed on a large diversity of fungi and can help plant growth by preferentially grazing on pathogenic soil fungi [205]. In many cases, the effects of invertebrate mycophagous grazers on plant communities vary among field and laboratory experiments [49]. 

Overall, mycophagous invertebrates can have a large impact on agriculture. The biocontrol potential of these invertebrates on pathogenic fungi has not been fully explored yet despite some species showing promising effects [206,207,208]. Invertebrates could be used as a more environmentally friendly alternative to fungicides, but their efficacy and viability still need more research. Finally, there may be some potential for the European truffle industry to exploit invertebrates in dispersing inoculum [170].

## 5. Conclusions

Mycophagy is a widespread phenomenon present in many invertebrate species. It has implications in many ecosystems and agriculture, but current research has only scratched the surface of these interactions. Many studies already performed lack a large scope or practical applications. New uses for techniques such as DNA metabarcoding can help give an idea of the diversity of organisms present in these interactions. The results of our literature search give a small insight into the diversity of mycophagous invertebrates and the fungi they feed on. We are aware that our literature search was non-exhaustive. Nonetheless, we were able to observe some general trends, such as the higher taxonomic groups that have thus far been most studied (Agaricomycetes for fungi, Coleoptera and Diptera for invertebrates). In addition, our work reveals important research questions for future work on this topic:

− How host-specific are invertebrates that utilize sporocarps as an environment for reproduction and development?
− What is the impact of spore dispersal on fungal communities?
− How do spore characteristics such as wall thickness and melanization affect viability after digestion?
− What is the difference between invertebrates and mammals in their spore dispersal efficacy?
− Does grazing by invertebrates have a larger effect on fungal communities?
− How do fungal secondary metabolites impact invertebrate communities?
− How do fungal secondary metabolites affect the behavior of invertebrates?
− Can mycophagous invertebrates be used as a suitable replacement for fungicides in agriculture?

## Data Availability

The data presented in this study are available in Table S1.

## Supplementary Materials

The following supporting information can be downloaded at: https://www.mdpi.com/article/10.3390/jof9020163/s1, Table S1: All 6093 observations of invertebrate mycophagy resulting from a Web of Science literature search.
